# Modest rescue of RBFOX1 splicing function attenuates Huntington’s disease features

**DOI:** 10.1186/s10020-026-01471-y

**Published:** 2026-04-02

**Authors:** David Lozano-Muñoz, Ainara Elorza, Miriam Lucas-Santamaría, María Santos-Galindo, Alberto Parras, José J. Lucas

**Affiliations:** 1 Center for Molecular Biology Severo Ochoa (CBM Severo Ochoa) CSIC/UAM, C/ Nicolás Cabrera, 1, Madrid, 28049 Spain; 2 Networking Biomedicine Research Center on Neurodegenerative Diseases (CIBER-NED), Instituto de Salud Carlos III, Madrid, 28031 Spain

**Keywords:** Huntington´s disease, Splicing, RBFOX, A2BP1, Transgenic mice

## Abstract

**Background:**

RNA mis-splicing underlies a growing number of neurological disorders and, consequently, splicing correction therapies have been developed for some monogenic forms, like spinal muscular atrophy or neuronal ceroid lipofuscinosis. In Huntington’s disease (HD), alternative splicing alteration emerged as a molecular mechanism in view of individually reported mis-splicing events in neurodegeneration-linked genes such as *HTT* itself, *MAPT* and *TAF1*. Later, more systematic genome-wide RNA-seq analyses of HD brains revealed mis-splicing signatures involving additional neurodegeneration-linked genes. Individual correction of each of the potentially pathogenic mis-spliced genes would be unapproachable. However, the identification of upstream pivotal splicing factors altered in HD may be useful to design pleiotropic therapeutic strategies. We previously performed motif-enrichment analyses of the sequences flanking exons that are mis-spliced in HD and identified RBFOX splicing factors as underlying candidates.

**Methods:**

We performed RT-PCR and Western blot analyses of RBFOX in post-mortem brain samples from HD patients and mice. We generated transgenic mouse lines overexpressing RBFOX1 in forebrain neurons and performed RNA-seq to analyze its impact on HD-associated mis-splicing. In addition, we combined HD mice with RBFOX1-overexpressing mice to verify correction of Rbfox1 levels and mis-splicing of RBFOX target genes, and performed histopathological and motor behavioral analyses.

**Results:**

We observed that decreased expression of Rbfox1 in striatum of HD mice at early stages of disease progression correlates with a reduction of Rbfox1 immunostaining particularly in the nucleus. This prompted us to generate transgenic mouse lines overexpressing the nuclear isoform of RBFOX1. The overexpression of RBFOX1 in this new transgenic mouse line induced widespread alternative splicing changes that significantly overlapped with genes mis-spliced in brains of both HD patients and mouse models. We found that moderate neuronal RBFOX1 overexpression in HD mice results in correction of several HD-associated mis-splicing events and in attenuation of neurodegeneration and motor symptoms.

**Conclusions:**

These results demonstrate that the observed decrease of RBFOX1 levels in brains of HD patients and mice contributes to HD pathogenesis and suggest therapeutic potential of RBFOX-increasing strategies for HD.

**Supplementary Information:**

The online version contains supplementary material available at 10.1186/s10020-026-01471-y.

## Introduction

Alternative splicing (AS) enables the generation of multiple mRNAs and protein isoforms from a single gene, playing a crucial role in increasing biological complexity. AS regulatory networks are essential for maintaining normal physiological functions, and mis-splicing has been proposed to play a role in brain pathologies, including neurodegenerative diseases like Alzheimer´s disease (Raj et al. 2018, Hsieh et al. 2019), amyotrophic lateral sclerosis (Luisier et al. 2018, Fratta et al. 2018) or Huntington’s disease (HD) (Lin et al. 2016, Schilling et al. 2019, Elorza et al. 2021).

HD is an autosomal dominant neurodegenerative disorder characterized by motor abnormalities together with psychiatric and cognitive symptoms, due to progressive brain atrophy, particularly affecting the striatum and cerebral cortex (Walker 2007). HD is caused by a CAG repeat expansion in exon 1 of the *Huntingtin* (*HTT*) gene, encoding a polyglutamine (polyQ) stretch in the N-terminal region of the HTT protein (HDCRG 1993) with a well-documented pathogenic role (Bates et al. 2015). Similar polyQ-encoding CAG triplet repeats expansions cause eight additional neurological disorders, such as some spinocerebellar ataxias (SCAs) and spinal-bulbar muscular atrophy (SBMA) (Orr and Zoghbi 2007). Beyond polyQ-driven toxicity, increasing evidence indicates that expanded CAG triplet-containing mRNAs cause additional toxicity in these and other triplet repeat disorders such as myotonic dystrophy type 1 (DM1) (Li et al. 2008, Ranum and Cooper 2006). Particularly, it has been suggested an aberrant direct interaction of the expanded CAG triplet repeat containing mRNAs with certain splicing factors (SFs) such as MBNL1 (Mykowska et al. 2011), U2AF2 (Schilling et al. 2019, Tsoi et al. 2011) and SRSF6 (Schilling et al. 2019).

The detection of alterations in the AS machinery in HD was followed by the identification of individual mis-splicing events that could significantly contribute to HD pathogenesis, such as an intron retention in *HTT* that favors a highly toxic exon1-encoded form of the protein (Sathasivam et al. 2013), the increased inclusion of *MAPT* exon 10 leading to a detrimental increase in four-tubulin binding repeat-tau (Fernandez-Nogales et al. 2014) and the use of an AS site in exon 5 of *TAF1* that correlates with decreased expression of this transcription factor (Hernandez et al. 2020), as in X-linked dystonia parkinsonism (Makino et al. 2007, Diaw and Lohmann 2020).

Apart from the mentioned individually identified pathogenic mis-splicing events, through a human/mouse intersect RNA-seq analysis, we were able to define a genome-wide striatal mis-splicing signature triggered by the HD-causing mutation in both mice and humans (Elorza et al. 2021, Xing et al. 2021), which allowed us to identify additional likely effectors in HD pathogenesis. More precisely, we found six neurodegeneration-linked genes whose mis-splicing correlates with their decreased protein levels. These are: *CCDC88C* (linked to SCA40, OMIM #616053), *KCTD17* linked to myoclonic dystonia 26 (DYT26) OMIM #616398], *SYNJ1* [linked to early onset Parkinson disease 20 (PARK20), OMIM #615530], *VPS13C* [linked to early onset Parkinson disease 23 (PARK23), OMIM #616840], *TRPM7* [linked to amyotrophic lateral sclerosis-parkinsonism/dementia complex (ALSP/DC), OMIM #105500] and *SLC9A5* [which has been suggested to be linked to episodic kinesigenic dyskinesia 2 (EKD2)] (Elorza et al. 2021).

Correction of each of the aforementioned potentially pathogenic mis-splicing events might be therapeutically relevant, but approaching them individually would be inefficient and unaffordable. Alternatively, the identification of pivotal upstream SFs altered in HD may lead to the development of therapeutic strategies to simultaneously amend multiple neurodegeneration-associated mis-spliced genes. In this context, our prior bioinformatics analysis of the sequences flanking mis-spliced exons in HD revealed that the binding motif of the RBFOX family of SFs [(U)GCAUG] was among those enriched in the flanking regions. Notably, the RBFOX family, whose proteins levels are decreased in brains of HD patients and mice, was the only one identified that does not bind U-rich motifs (Elorza et al. 2021).

The RBFOX family is constituted by three proteins, RBFOX1 (also known as Ataxin-2 binding protein 1, A2BP1), which is expressed selectively in neurons and in heart and skeletal muscle; RBFOX2 (RBM9), with a wider expression pattern (whole embryo, ovary, stem cells, brain and skeletal muscle); and RBFOX3 (NeuN), which is expressed exclusively in neurons (Kuroyanagi 2009, Kim et al. 2009). Furthermore, the protein levels of these three SFs are decreased in brains of HD patients and mice (Elorza et al. 2021), and we reasoned that correcting their decrease might be beneficial for HD and that this could be tested by generating mice with increased expression of any of the members of the family.

Here, we generated RBFOX-overexpressing mice and demonstrate that compensating for the reduction of Rbfox1 observed in HD mouse models attenuates their molecular, histological, and behavioral phenotypes.

## Materials and methods

### Human brain tissue samples

Brain specimens used in this study from striatum of HD patients and controls for IHC and RT-qPCR were provided by Institute of Neuropathology Brain Bank (Hospitalet de Llobregat, Spain), the Neurological Tissue Bank of the IDIBAPS Biobank (Barcelona, Spain), Banco de Tejidos Fundación Cien (Madrid, Spain) and the Netherlands Brain Bank (Amsterdam, The Netherlands). Control samples were selected from those available at the corresponding brain bank to best match the HD cases in terms of age, sex, and postmortem interval. Written informed consent for brain removal after death for diagnostic and research purposes was obtained from brain donors and/or their next of kin. Procedures, information and consent forms were reviewed and approved by the Bioethics Subcommittee of Consejo Superior de Investigaciones Científicas (CSIC, Madrid, Spain).

### Mice

R6/1 transgenic mice for the human exon-1-Htt gene (Mangiarini et al. 1997) were maintained in B6CBAF1 background (CAG repeat length between 150 and 170). CamKII-tTA mouse lines (Mayford et al. 1996) were maintained in a pure C57BL/6J background. Mice with transgenic expression of human RBFOX1 under a tetracycline-regulated promoter (TgRBFOX1) were generated for this study (for details see ‘Generation of TgRBFOX1’ below) and were maintained in C57BL/6J background. All animals were maintained in the animal facility of Centro de Biología Molecular Severo Ochoa (CBMSO). Mice were housed in a maximum of five per cage with food and water available ad libitum, and maintained in a temperature-controlled environment with a normal 12-hour light/dark cycle with light onset at 8:00 am. Mice breeding, housing and behavioral tests followed the guidelines of the Council of Europe Convention ETS123, and were performed according to protocols approved by the CBMSO Institutional Animal Care and Utilization Committee (Comité de Ética de Experimentación Animal del CBM, CEEA-CBM), CSIC’s Ethics Committee and Comunidad de Madrid Regional Government protocols PROEX 293/15 and PROEX 247.1/20.

### Generation of RBFOX1 transgenic mice

Human RBFOX1 cDNA (from pENTR-A2BP1, Addgene, 16176) was independently cloned into a plasmid containing a bidirectional TetO sequence that also harbors the β-Gal reporter with a NLS (pBI-G, Clontech, 631004). The resulting construct was linearized and microinjected into single‐cell C57BL/6JxCBA embryos. This resulted in the generation of β-Gal-BiTetO-hRBFOX1 founder mice that were subsequently backcrossed into a pure C57BL/6J background. All the experiments were performed between the 6th and the 11th backcross to C57BL/6J background. Mice were then crossed with CamKII-tTA mice in C57BL/6J background to obtain conditional double transgenic mice with forebrain neuronal expression of hRBFOX1 (TgRBFOX1).

### Open field test

Locomotor activity was assessed in 27.5 cm x 27.5 cm clear open-field boxes equipped with photo-beam detectors to monitor movement. Activity was recorded with a MED Associates’ Activity Monitor (MED Associates) and were analyzed with the MED Associates’ Activity Monitor Data Analysis v.5.93.773. Mice were placed in the center of the open-field apparatus and allowed to move freely during 15 min.

### Rotarod test

Motor coordination was assessed with the accelerating rotarod apparatus (Ugo Basile, Comerio, Italy). Mice underwent training over two consecutive days: on the first day, they performed four 1-min trials at a constant speed of 4 rpm; on the second day, they completed four 2-min trials, with the first min accelerating from 4 rpm to 8 rpm and the second min at constant 8 rpm. On the third day, the test phase was conducted with the rotarod accelerating from 4 to 40 rpm over 5 min. The latency to fall from the rotarod was calculated as the mean latency across four accelerating trials and normalized to performance at 8 weeks of age, when no motor impairment was observed.

### Grip strength test

Forelimb strength was assessed using the grip strength test (Bioseb). Mice were held by the tail and allowed to grasp the grid of the apparatus with their forelimbs. Once they had firmly grasped the grid, they were gently pulled away. The maximum force exerted before releasing the grid was recorded by the apparatus. Each mouse underwent four trials, and the average value was used for the final analysis.

### Tissue preparation for staining

For human samples, formalin-fixed, paraffin-embedded tissue from striatum was used. Sections (5-µm thick) were mounted on superfrost-plus tissue slides (Menzel-Gläser) and deparaffinized. For mouse samples, mice were sacrificed using CO_2_. Brains were quickly extracted and the left hemisphere was immersed in 4% paraformaldehyde overnight. After profuse washing in phosphate-buffered saline (PBS), hemispheres were immersed in sucrose 30% in PBS for a minimum of 72 h and then embedded in OCT (Optimum Cutting Temperature compound, Tissue-Tek, Sakura Finetek Europe, ref. 4583), frozen and stored at -80 °C until use. Mouse sagittal Sect.  (30 μm thick) were cut sequentially on a cryostat (Thermo Scientific), collected and stored free-floating in glycol-containing solution (30% glycerol, 30% ethylene glycol in 0.02 M phosphate buffer) at -20 °C.

### Immunohistochemistry

For human samples, peroxidase activity was quenched with 0.3% H_2_O_2_ in methanol for 30 min, followed by antigen retrieval with 10 mM pH 6.0 citrate buffer heated in microwave for 15 min. For mouse sections, tissues were first washed in PBS and then immersed in 0.3% H_2_O_2_ in PBS for 45 min to quench endogenous peroxidase activity. After PBS-washes, sections were immersed for 1 h in blocking solution (PBS containing 0.5% fetal bovine serum, 0.3% Triton X-100 and 1% bovine serum albumin) and incubated overnight at 4 °C with primary antibody diluted in blocking solution. After washing, brain sections were incubated first with biotinylated goat anti-rabbit or anti-mouse secondary antibodies and then with avidin-biotin complex using the Elite Vectastain kit (Vector Laboratories, PK-6101 and PK-6102). Chromogen reactions were performed with diaminobenzidine (SIGMAFAST DAB, Sigma, D4293) for 10 min. Mouse sections were mounted on glass slides and coverslipped with Mowiol (Calbiochem, Cat. 475904), while human sections were first dehydrated and then mounted with DePex (SERVA). Images were captured using an Olympus BX41 microscope with an Olympus camera DP-70 (Olympus Denmark A/S).

Antibodies: rabbit anti-Rbfox1 (1:5000, Novus, NBP1-90304), rabbit anti-β-Gal (1:2000, Invitrogen, A-11132), rabbit Darpp32 (1:3000, BD, 611520) and rabbit Cleaved caspase-3 (1:100, Cell Signaling, 9661) for mouse samples. Mouse anti-RBFOX1 (1:5000, Merk Millipore, MABE985) for human samples.

### Quantification of cleaved caspase-3-positive cells

The total number of immunopositive cells in three sagittal sections (lateral coordinates 1.20, 1.44 and 1.68 mm) was quantified for each animal using an Olympus BX41 microscope with an Olympus camera DP-70 (Olympus Denmark A/S). Mean values per genotype were used for statistical comparison.

### Striatal area measurement

The striatal area was measured by Darpp32-positive immunostaining in sagittal Sect.  (2 sections/animal) (lateral coordinate 2.64 and 2.16 mm). Images were captured at 2.5x magnification (Canon EOS 450D digital camera) and area quantification was made using ImageJ software. Mean values for each genotype were used for statistical comparison.

### Immunofluorescence

Mouse brain sections were first washed in phosphate buffer (PB) and subsequently blocked for 1 h at room temperature with a solution containing 3% normal goat serum, 0.1 M PB, and 0.1% Triton X-100. Sections were then incubated overnight at 4 °C with the primary antibody (rabbit anti-RBFOX1 Novus, NBP1-90304, 1:1000) diluted in the same blocking solution. After incubation with the primary antibody, sections were washed and incubated for 1 h at room temperature with secondary fluorophore-conjugated antibodies (donkey anti-mouse Alexa Fluor 488, 1:500; donkey anti-rabbit Alexa Fluor 488, 1:500). Nuclei were counterstained with DAPI (1:10,000) for 10 min. Sections were mounted on glass slides using ProLong™ Gold Antifade Mountant (ThermoFisher Scientific).

Images were acquired using a Zeiss LSM800 inverted confocal microscope and ZEN software (version 3.3). Image deconvolution was performed with Huygens software (version 24.10, SVI). For signal quantification, nuclear masks were generated using the DAPI channel with Cellpose plugin, and mean gray value was measured using Fiji (ImageJ).

### Protein level analysis by Western blot

Mouse brains were rapidly dissected on an ice-cold surface, and the different brain structures were stored at -80 °C. Protein extracts were prepared by homogenizing brain structures in ice-cold extraction buffer (20 mM HEPES pH 7.4, 100 mM NaCl, 20 mM NaF, 1% Triton X-100, 1 mM sodium orthovanadate, 1 µM okadaic acid, 5 mM sodium pyrophosphate, 30 mM β-glycerophosphate, 5 mM EDTA, protease inhibitors [Complete, Roche, Cat. No 11697498001]). Homogenates were centrifuged at 14,100 x g for 15 min at 4 °C. The resulting supernatant was collected, and protein content was determined by the Quick Start Bradford Protein Assay (Bio-Rad, 500 − 0203). 15 µg of total protein were electrophoresed on an 8–10% SDS-polyacrylamide gel, transferred to a nitrocellulose blotting membrane (Amersham Protran 0.45 μm, GE Healthcare Life Sciences, 10600002) and blocked in Tris-buffered saline with Tween 20 (TBS-T) (150 mM NaCl, 20 mM Tris–HCl, pH 7.5, 0.1% Tween 20) supplemented with 5% non-fat dry milk. Membranes were incubated overnight at 4 °C with primary antibody in TBS-T supplemented with 5% non-fat dry milk, washed with TBS-T and then incubated with HRP-conjugated anti-mouse IgG (1:2000, DAKO, P0447) or anti-rabbit IgG (1:2000, DAKO, P0448). Signals were detected using the ECL detection kit (PerkinElmer, NEL105001EA).

Antibodies: mouse anti-β-actin (1:25000, Sigma, A2228), mouse anti-Rbfox1 (1:2000, Merck Millipore, MABE985), rabbit anti-Rbfox1 (1:1000, Novus, NBP1-90304), mouse anti-Rbfox2 (1:1000, Abcam, ab264154) and mouse anti-Rbfox3 (1:2000, Merck Millipore, MAB377) for mouse samples.

### RNA sequencing and analysis

Mice were sacrificed using CO_2_ and mouse brains were quickly dissected on an ice-cold plate and the different structures stored at -80 °C. Total RNA from forebrain of P5 WT (*n* = 3) and TgRBFOX1 (*n* = 3) mice was isolated using the Maxwell^®^ 16 RSC simplyRNA Tissue Kit (Promega, AS1340). Total RNA was quantified by Qubit^®^ RNA BR Assay kit (Thermo Fisher Scientific) and the RNA Quality number (RQN) was estimated using the Qsep100TM (BiOptic) detecting, on average, a RQN value over 9. Stranded poly(A) enriched libraries were generated and sequenced on a Novaseq X Plus (Novogene) with a read length of 2 × 150 bp, generating at least 100 million reads per sample.

The quality of FASTQ files was assessed using FastQC v0.11.9. For rMATS analysis, sequencing reads in FASTQ format were aligned to the mouse reference genome (mm10; GRCm38.100) using STAR v2.7.10a (Dobin et al. 2013). The resulting BAM files were analyzed with rMATS, and splicing events with more than five reads, an absolute ΔPSI greater than 15, and an FDR below 0.05 were considered differentially spliced.

### Enrichment analysis of RBFOX targets among HD signature

The overlap between the HD mis-splicing signature and RBFOX1 direct targets (Weyn-Vanhentenryck et al. 2014) was assessed using a hypergeometric test implemented in R. The representation factor was calculated as the ratio between the observed number of overlapping genes and the expected number of overlapping genes assuming random sampling from two independent gene sets.

### Reverse transcriptase quantitative and semiquantitative PCR

Retrotranscription reactions were performed using iScript™ Reverse Transcription Supermix for RT-qPCR (Bio-Rad). Relative quantification was carried out for mRNA analysis in striatum of mice (WT and R6/1). qRT-PCR (CFX 384 Biorad) was carried out with 5 ng of cDNA in a volume of 4 µl with 1 µl of 5 µM forward and reverse primers mix and 5 µl of SsoFast EvaGreen Supermix premix (Biorad, CN172-5204). Triplicate reactions were carried out for each mRNA. The following amplification protocol was used: initial denaturation of 5 s at 95 °C + 40 cycles x [5s at 95 °C + 5 s at 60 °C] + [5 s at 60 °C + 5 s at 95 °C]. Fluorescence was taken at the end of elongation step. Data were analysed by GenEx 5.3.7 software (Multid AnaLyses AB). The mRNA levels were normalized first relative to total RNA and then relative to the 18 S ribosome subunit, β-ACTIN, GAPDH and β-TUBULIN gene expression in each sample.

Selected mis-spliced HD events were evaluated with semiquantitative reverse transcription-PCR. cDNA (50 ng) was amplified with specific primers. PCR products were resolved on 2% resolution Metaphor agarose gels (Lonza).

### Primers

The sequences of all primers used in this study are detailed in the following table:

**Table:** 

	Forward	Reverse
Human		
RBFOX1	5’-GCCACAGCACGTGTAATGACA-3’	5’-CCACAACTGGATTCAATTTCCAG-3’
18s	5′-ATCCATTGGAGGGCAAGTC-3′	5′-GCTCCCAAGATCCAACTACG-3′
β-TUBULIN	5′-CTTTGTGGAATGGATCCCCA-3′	5′-GACTGCCATCTTGAGGCCA-3′
β-ACTIN	Cat. No. qA-01–0104 S (TATAA)	Cat. No. qA-01–0104 S (TATAA)
*Human-mouse* RBFOX1	5’-GCCACAGCACGTGTAATGACA-3’	5’-CCACAACTGGATTCAATTTCCAG-3’
*Mouse*		
Rbfox1	5′- GACCCCTACCACCACACACT − 3′	5′- TCTTGGCATCGGTCAAGG − 3′
Gapdh	5′-CTCCCACTCTTCCACCTTCG-3′	5′-CATACCAGGAAATGAGCTTGACAA − 3′
β-Actin	5’-CTAAGGCCAACCGTGAAAAG-3’	5’-ACCAGAGGCATACAGGGACA-3’
18s	5’-CTCAACACGGGAAACCTCAC-3’	5’-CGCTCCACCAACTAAGAACG-3’
Synj1	5’-CCCAGACTCTAGAGCCCAAGA-3’	5’-GCTTGAGGGGAAGGCTGATTAC-3’
Slc9a5	5′-GCTGAGGGTGAAGAGGAGTGA-3′	5′- GCTGATGGCATCTCGGATGTT-3′
MacF1	5′-CAGCAGGTGTGGCTGTTAGC-3′	5′-CCAGACATCAAAGTCAAAGTTGGC-3′
Cadps	5′-ACTGCAACAAAACGAGGAGCA-3′	5′-TGCATCCATGTCCACTGCAAA-3′
Vps13c	5′-CCCAGACTCTAGAGCCCAAGA-3′	5′-CCCAGACTCTAGAGCCCAAGA-3′
Plch1	5′- CTACCGGCATGTCTACCTGGA-3′	5′- TTTCTGAAGAAGCGTGCCTGG-3′
Qrich1	5′-GGTAGAACGGAGGCAGCGG-3′	5′- TTCCTTCATCCTGTGTTGCGG-3′

### Statistical analysis

Details regarding the statistical tests and sample sizes are indicated in the figure legends or in the “materials and methods” section. Blinding was performed during data collection and analysis. Statistical analysis and graphs generation were performed using R software with the ‘ggplot2’ package.

Data are represented as mean ± SEM (Standard Error of the Mean), unless otherwise indicated. Normality of data distribution was assessed using the Kolmogorov-Smirnov test, and homogeneity of variances was evaluated with Levene’s test. For comparisons between two independent groups, either a two-tailed Student’s t-test (for normally distributed data) or Mann-Whitney U-test (for non-normally distributed data) was performed. For multiple group comparisons, data were analyzed by one-way ANOVA followed by a Tukey’s post-hoc test (for normally distributed data) or by Games-Howell post hoc test (for non-normally distributed data). The threshold for statistical significance was set at *p* < 0.05, or lower where specified.

Regarding the overlap between RBFOX targets and HD mis-splicing signature, enrichment analyses were carried out with hypergeometric test. Background dataset for analysis was generated with only one-to-one orthologues with sufficient read coverage detected by previous RNA-seq in human and mouse (*n* = 12,882).

### Data availability

Data generated in this study are available from the corresponding author upon reasonable request. The RNA-seq dataset will be available at the European Nucleotide Archive (ENA) database with accession number PRJEB110930.

## Results

### Early decrease of Rbfox1 protein levels in HD mice

**Fig. 1:**
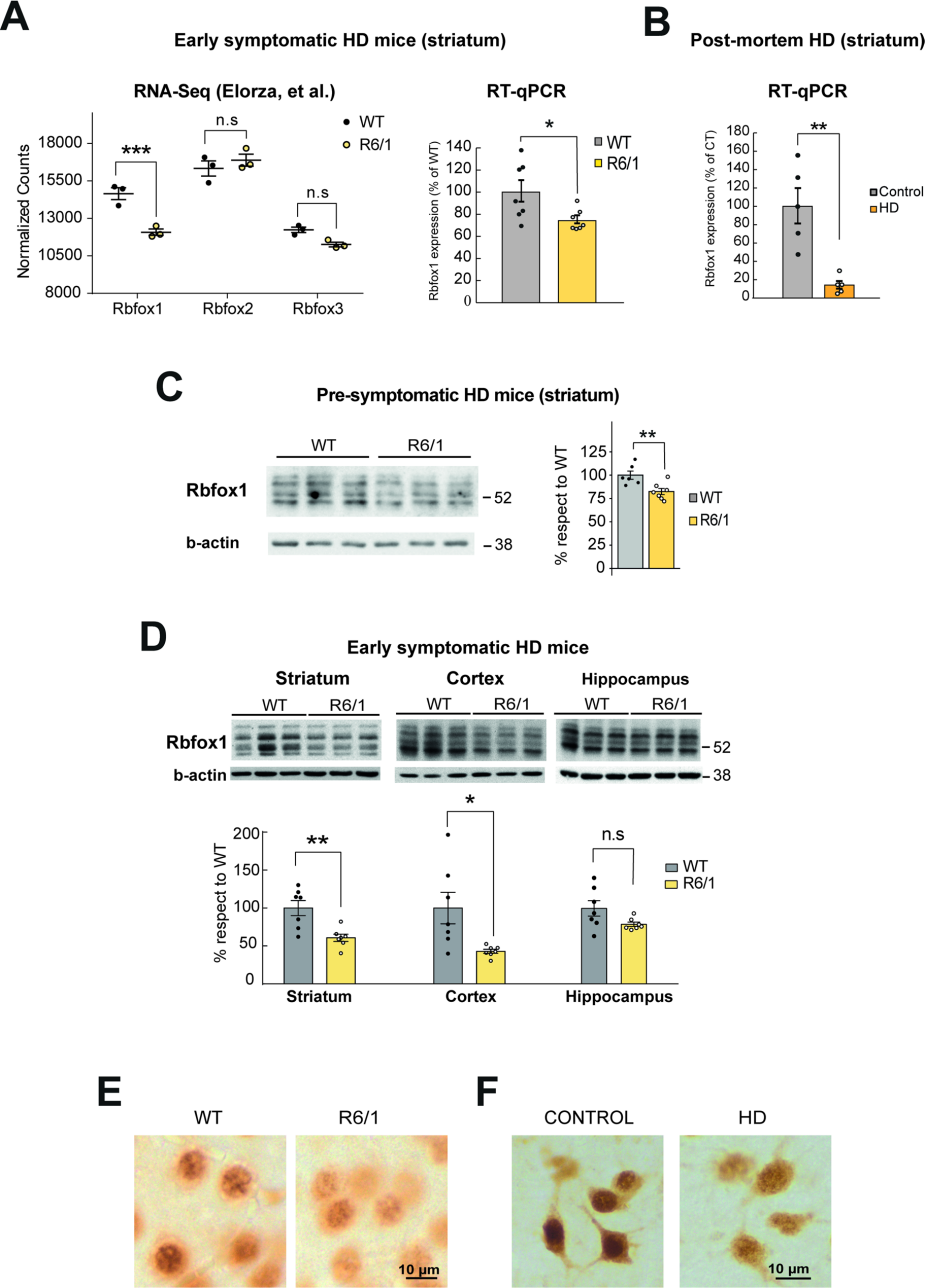
Early decrease of Rbfox1 protein levels in HD mice. **A** Normalized counts of the three Rbfox genes in striatal RNA from 3.5-month-old WT (*n* = 3) and R6/1 (*n* = 3) mice, according to RNA-seq datasets from Elorza et al., 2021 (Wald test with Benjamini–Hochberg correction for multiple testing; ***FDR < 0.001). Quantification of Rbfox1 transcript levels by RT-qPCR in striatum from 3.5-month-old WT (*n* = 7 [4 males and 3 females]) and R6/1 (*n* = 7 [4 males and 3 females]) mice, **B** and in striatum from human control (*n* = 5) and HD post-mortem cases (*n* = 5). **C** Western blot with the 1D10 MABE985 antibody that recognizes multiple isoforms of the Rbfox1 protein in the striatum of 1.5-month-old WT (*n* = 6 [3 males and 3 females]) and R6/1 (*n* = 7 [4 males and 3 females]) mice, with quantification normalized to β-actin. **D** Rbfox1 protein levels analyzed by Western blot also with the 1D10 MABE985 antibody in striatum, cortex, and hippocampus of 3.5-month-old WT (*n* = 7 [4 males and 3 females]) and R6/1 (*n* = 7 [4 males and 3 females]) mice, and also normalized to β-actin. Data represent mean ± SEM. (Student’s t-test or Wilcoxon rank-sum test; **P < 0.01; *P < 0.05). **E**–**F** Representative RBFOX1 immunohistochemistry in the striatum of 3.5-month-old WT and R6/1 mice, and in the striatum of control and HD subjects. Scale bar = 10 μm

In order to generate RBFOX overexpressing mice, we first considered which RBFOX paralogue would be more relevant to the mis-splicing signature of HD. Interestingly, our previous data from RNA-seq analysis of the widely used R6/1 mouse model of HD at early symptomatic stages of disease (3.5 month-old) showed down-regulation of *Rbfox1* mRNA (FC = 0.8; *Padj =* 2,96 × 10^− 5^) (Elorza et al. 2021), while *Rbfox2* and *Rbfox3* did not differ between WT and R6/1 mice (Fig. 1A). Furthermore, we here corroborate the decrease in *Rbfox1* transcript levels by RT-qPCR in an independent set of samples from early symptomatic R6/1 mice (25.8% decrease, *P* = 0.03) (Fig. 1A) and in postmortem brain samples from HD cases (Fig. 1B).

Western-blot analysis of the striatum of pre-symptomatic R6/1 mice (1.5 months old) revealed a significant decrease of Rbfox1 protein levels (14.1%; *P* = 0.009), reinforcing downregulation of Rbfox1 as a possible early event in HD pathogenesis (Fig. 1C). Further analysis revealed that the reduction in Rbfox1 protein levels observed in the striatum of early-symptomatic R6/1 mice (39.3% decrease, *P* = 0.004) was also present in the cortex (57.1% decrease, *P* = 0.016), a brain region notably affected in HD. A similar decrease is observed with a different anti-Rbfox1 antibody that seems specific for one of the splicing isoforms of Rbfox1, as it only detects the 54 kDa band (Supplementary Fig. 1). In contrast, no significant reduction in Rbfox1 levels was observed in the hippocampus, a region moderately impacted by the disease (Fig. 1D).

Together, these pieces of evidence pointed to Rbfox1 as the member of the family that is earliest altered and therefore best candidate to, upon overexpression, correct mis-splicing and pathology in HD mice. Regarding the different RBFOX1 isoforms, we chose to overexpress the RBFOX1 transcript isoform that lacks the alternatively spliced exon A53, as this preserves the nuclear localization signal required for RBFOX1 to act as a SF (Damianov and Black 2010, Lee et al. 2009, Nakahata and Kawamoto 2005), in contrast to the cytoplasmic forms of RBFOX1 involved in mRNA stability and translation (Lee et al. 2016). Furthermore, we verified by immunohistochemistry staining in striatal sections from R6/1 mice and HD patients that the decrease in RBFOX1 protein previously observed by Western blot predominantly corresponds to nuclear forms (Fig. 1E-F).

### Generation of Tg mouse line with conditional neuronal overexpression of RBFOX1

**Fig. 2:**
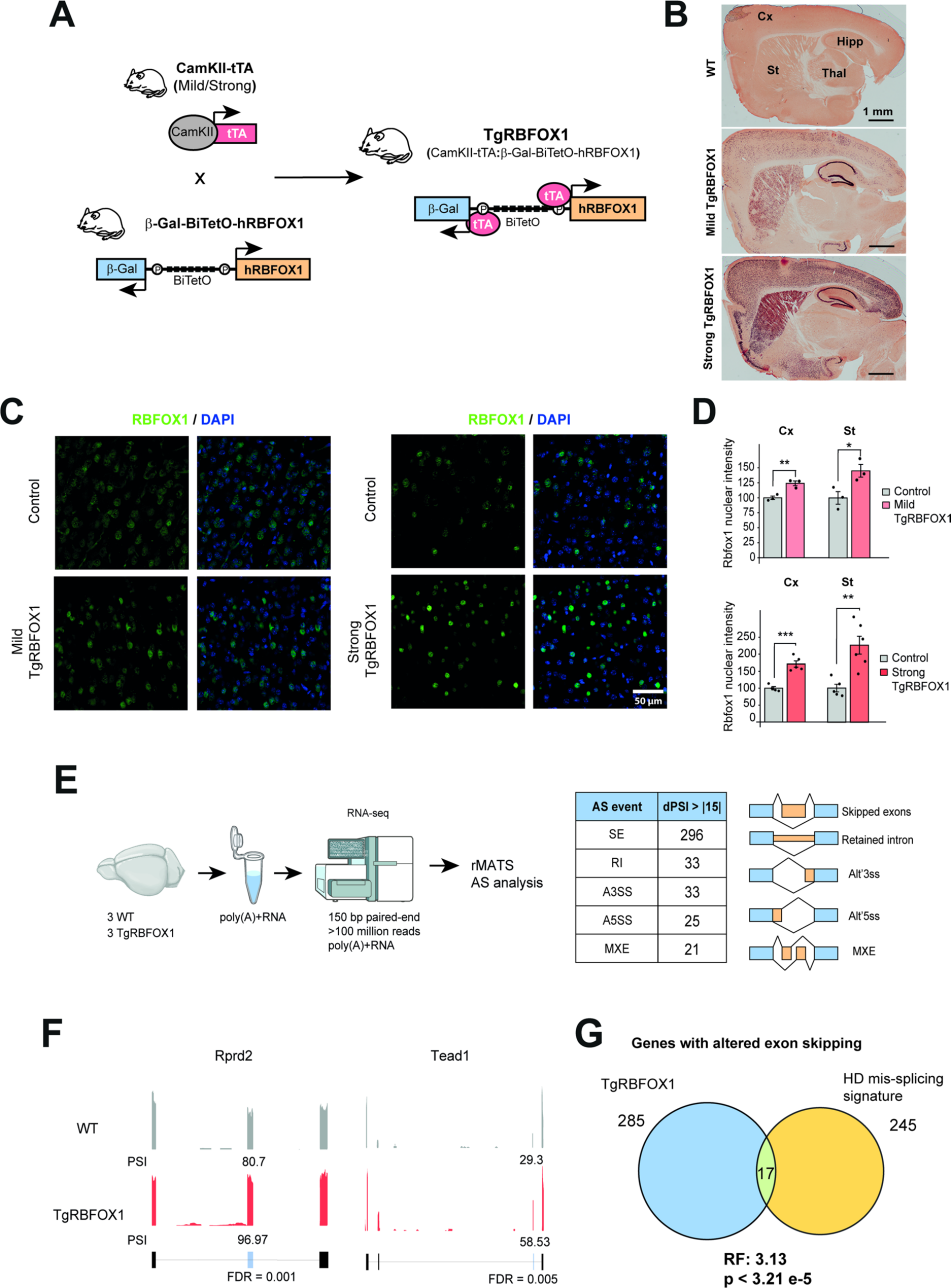
Generation of transgenic mice with conditional overexpression of RBFOX1 nuclear isoform in forebrain neurons. **A** Mice expressing tTA under control of the CamKII promoter (CamKII-tTA mice [Mild/Strong]) were bred with mice carrying the β-Gal-BiTetO-RBFOX1 construct to yield TgRBFOX1 (CamKII-tTA:β-Gal-BiTetO-RBFOX1) mice. **B** Immunohistochemistry with anti-β-Gal antibody in sagittal sections from 1.5 month-old WT and TgRBFOX1 mice. Scale bar = 1 mm. **C** Representative immunofluorescence images showing RBFOX1 signal in cortex of control and TgRBFOX1 generated with the ‘Mild’ or ‘Strong’ CamKII-tTA driver line. Scale bar: 50 μm. **D** Quantification of nuclear RBFOX1 intensity signal detected by immunofluorescence in cortex and striatum of TgRBFOX1 mice generated using the ‘Mild’ or ‘Strong’ CamKII-tTA driver lines. Groups: Control (*n* = 3 [2 males and 1 female];5 [3 males and 2 females]), MildTgRBFOX1 (*n* = 3 [1 male and 2 females]), and StrongTgRBFOX1 (*n* = 5 [3 males and 2 females]) (Student´s t test; *P < 0.05; **P < 0.01; ***P < 0.001). Data represent mean ± SEM. **E** RNA-seq was performed on forebrain of control (*n* = 3) and TgRBFOX1 1.5 month-old mice (*n* = 3), and splicing was analyzed using rMATS. The different types of alternative splicing events with an absolute dPSI > 15 and FDR < 0.05 between control and TgRBFOX1 mice are indicated. **F** Representative Integrative Genomics Viewer profiles from RNA-seq data of a control and a TgRBFOX1 mouse illustrate the changes in inclusion levels of the indicated RBFOX target exons. PSI values of alternative exons and FDR values from rMATS analysis are indicated. **G** Venn diagram showing the intersection between the 245 genes with exons mis-spliced in HD and the 285 genes with splicing changes in exons in TgRBFOX1 mice. Representation factor (RF) and p-value were determined with hypergeometric test, using as background the human-mouse orthologous genes coincidentally detected in the human and mouse RNA-seq datasets used to define the HD mis-splicing signature (*n* = 12,882)

Given that mutations in Rbfox1 lead to neurodevelopmental disorders (Bill et al. 2013), to avoid confounding neurodevelopmental artifacts, we decided to use the double transgenic tTA (Tet-Off) conditional system that allows to generate mice with different time windows and levels of transgene expression. With this purpose, we generated a β-Gal-BiTetO-hRBFOX1 transgenic mouse line, harboring a bidirectional tetracycline-controlled promoter (BitetO) flanked by the sequence of the hRBFOX1 nuclear isoform on one direction, and the sequence of β-Galactosidase (β-Gal) on the other, the latter as a reporter of transgene expression (Fig. 2A). The BitetO promoter in β-Gal-BiTetO-hRBFOX1 mice is essentially silent and, to achieve transgene transactivation in forebrain regions affected in HD —such as the striatum and the cortex—, we bred these mice with two different CamKII-tTA mouse lines that express the transactivator tTA in forebrain neurons. One of these mouse lines shows higher tTA expression, which starts at late embryonic stages (Parras et al. 2018), while the other shows milder, and only postnatal (Olla et al. 2023), expression.

The double transgenic mice, termed TgRBFOX1, express hRBFOX1 at different levels and time points, according to the specific CamKII-tTA driver mouse line used to generate them. TgRBFOX1 mice showed the expected neuronal pattern of transgene expression restricted to forebrain regions such as cortex, striatum and hippocampus, and the level was higher with the ‘Strong’ driver (versus the ‘Mild’ one), as evidenced by the detection of the reporter β-Gal (Fig. 2B). In good agreement, RT-qPCR analyses with primers specific for human Rbfox1 (h) and primers that equally bind the human and the mouse Rbfox1 sequence (h + m), confirmed overexpression of Rbfox1 transcript in both mouse lines, which was much higher in the StrongTgRBFOX1 line (Supplementary Fig. 2A-B). This was further confirmed by immunofluorescence of RBFOX1 followed by quantification of the nuclear signal intensity. As expected, the nuclear RBFOX1 signal also depended on the CamKII-tTA driver used, with Strong TgRBFOX1 mice showing higher levels compared to MildTgRBFOX1 mice in both in striatum and cortex (Fig. 2C-D).

Higher transgene expression correlated with detectable microcephaly (9.8% decrease in brain weight, *P* = 1.4 × 10 − 3), specifically in the StrongTgRBFOX1 line (Supplementary Fig. 2C). This prompted us to explore whether the MildTgRBFOX1 line showed signs of subtler anatomical alteration. However, immunohistochemical analysis with the striatal marker DARPP32 reveled normal striatal area in MildTgRBFOX1 mice (Supplementary Fig. 2D). A general behavioral characterization revealed that both MildTgRBFOX1 and StrongTgRBFOX1 mice were indistinguishable from wild type mice in the open field and the rotarod tests, at the two analyzed ages (2 and 3.5 moths) (Supplementary Fig. 3A-D). Furthermore, MildTgRBFOX1 mice were also indistinguishable from wild type mice in the grip strength test at the age of 5 months (Supplementary Fig. 3E).

Then we verified that RBFOX1 overexpression in TgRBFOX1 mice had an impact on AS. For that purpose, we performed RNA-seq analysis of 1.5-month-old StrongTgRBFOX1 mice. Analysis of differential splicing using rMATS revealed a total of 408 differential AS events in TgRBFOX1 mice, of which 296 corresponded to skipped exons (Fig. 2E) (Supplementary Table-S1) including direct RBFOX targets previously identified by individual-nucleotide resolution UV crosslinking and immunoprecipitation (iCLIP) (Weyn-Vanhentenryck et al. 2014) like *Rprd2* and *Tead1*, which showed increased inclusion upon RBFOX1 overexpression (Fig. 2F). We next compared the 285 genes with exons altered in TgRBFOX1 mice to the 245 genes previously found to be mis-spliced in the brains of both HD patients and early symptomatic R6/1 mice (Elorza et al. 2021), which revealed a statistically significant overlap (RF: 3.13, *P* < 3.21 × 10 − 5) (Fig. 2G and Supplementary Table-S2). More precisely, in terms of specific splicing events, seven are common and, as expected, most of these (five) change in opposite direction (Supplementary Table-S2).

### Overexpression of RBFOX1 attenuates HD-associated motor deficit and neuropathology of R6/1 mice

**Fig. 3:**
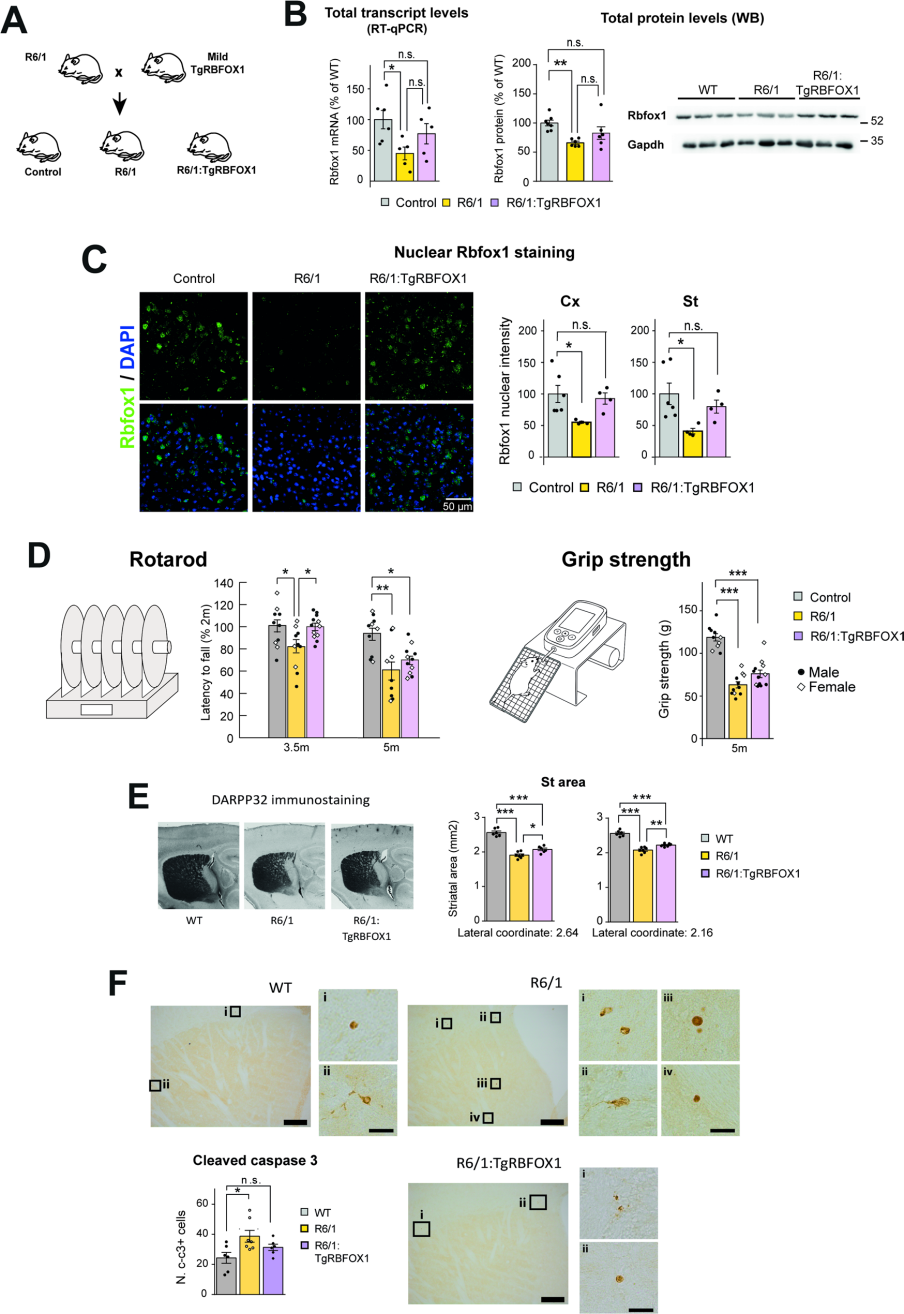
Overexpression of RBFOX1 delays HD-associated behaviors and neuropathology in R6/1 mice. **A** TgRBFOX1 mice line were bred with R6/1 mice to yield the three experimental genotypes: Control, R6/1 and R6/1:TgRBFOX1 **B** Total Rbfox1 transcript levels analyzed by RT-qPCR in the cortex of 3.5-month-old Control (*n* = 6 [3 males and 3 females]), R6/1 (*n* = 5 [3 males and 2 females]) and R6/1:TgRBFOX1 (*n* = 5 [2 males and 3 females]) mice. Protein levels analyzed by Western blot with the Novus antibody in the cortex of 3.5-month-old Control (*n* = 7 [4 males and 3 females]), R6/1 (*n* = 6 [3 males and 3 females]) and R6/1:TgRBFOX1 (*n* = 6 [3 males and 3 females]) mice, with quantification normalized to Gapdh. **C** Quantification of nuclear RBFOX1 intensity signal detected by immunofluorescence in cortex and striatum of control, R6/1 and R6/1:TgRBFOX1 mice generated using the ‘Mild’ CamKII-tTA driver line. Groups: Control (*n* = 6 [3 males and 3 females]), R6/1 (*n* = 4 [2 males and 2 females]), R6/1:TgRBFOX1 (*n* = 4 [2 males and 2 females]). **D** Evolution of the percentage of mean latency to fall in the accelerating rotarod test at 3.5 months and 5 months, normalized to the performance of each mouse at 2-months and forelimb strength in the grip strength test at 5 months in control, R6/1 and TgRBFOX1 (Control [6 males and 4 females]; R6/1 [6 males and 4 females]; and R6/1:TgRBFOX1 [7 males and 5 females]) mice at different ages. **E** Representative images of DARPP32-immunostained striatal (St) area and its quantification in sagittal sections at two different lateral coordinates of the mouse brain atlas for control (*n* = 6 [3 males and 3 females]), R6/1 (*n* = 7 [4 males and 3 females]) and R6/1:TgRBFOX1 (*n* = 6 [3 males and 3 females]) mice at 3.5 months of age. **F** Representative images showing immunostaining of cleaved caspase-3 and quantification of cleaved caspase-3-positive cells in control (*n* = 6 [3 males and 3 females]), R6/1 (*n* = 7 [4 males and 3 females]) and R6/1:TgRBFOX1 (*n* = 6 [3 males and 3 females]) mice Scale bars = 200 μm in large panels and 20 μm in the insets. Data represent mean ± SEM. Analysis of variance (ANOVA), followed by Tukey´s post-hoc test. (*P < 0.05, ** *P* < 0.01, *** *P* < 0.001, n.s.: not significant)

To explore whether correction of the RBFOX1 deficit can be beneficial to HD mice, we aimed to combine R6/1 and TgRBFOX1 mice. Since StrongTgRBFOX1 mice showed microcephaly (Supplementary Fig. 2C), we suspected that they were unlikely to correct HD-associated phenotypes of R6/1 mice. In fact, a pilot experiment revealed that motor coordination impairment in the rotarod test debuts earlier in R6/1 mice also harboring the StrongRBFOX1 transgenes than in plain R6/1 mice (Supplementary Fig. 4). For this study aiming correction of phenotypes of HD mice, we therefore decided to combine the HD mice with the MildTgRBFOX1 line (Fig. 3A), and the comparisons of the relevant resulting genotypes were performed on littermates (see exact genotypes and breeding schemes in Supplementary Fig. 5A). We first checked the degree of correction of RBFOX1 total transcript and protein levels. Although the difference between R6/1 and R6/1:TgRBFOX1 mice did not reach statistical significance, the significant deficits respect to wild type mice found in R6/1 mice, were no longer observed in R6/1:TgRBFOX1 mice (Fig. 3B). Similar results were obtained regarding nuclear RBFOX1 staining (Fig. 3C).

The accelerating rotarod and grip strength tests are among the most sensitive and widely used tests to evaluate the deficit in motor coordination and muscle strength in HD models (Menalled et al. 2014). Accordingly, they are commonly used to detect early behavioral deficits in R6 mice in preclinical testing (Hickey et al. 2005). For these reasons, we decided to subject WT, R6/1 and R6/1:TgRBFOX1 littermates to the accelerating rotarod and grip strength tests at ages at which R6/1 mice are known to show early (3.5 months) or advanced (5 months) phenotypes (Fig. 3D). Remarkably, we observed that overexpression of RBFOX1 attenuated the motor phenotype of R6/1 mice. More precisely, at the age of 3.5 months, R6/1:TgRBFOX1 mice performed better than R6/1 mice (Fig. 3D) and they still showed a tendency to perform better than R6/1 mice at the age of 5 months. In the grip strength test, a significant decrease was observed for R6/1 mice at 5 months and a trend toward phenotype attenuation was observed in R6/1:TgRBFOX1 mice, although the difference between R6/1 and R6/1:TgRBFOX1 did not reach statistical significance (Fig. 3D). As both rotarod performance and grip strength can be affected by body weight, mouse weights were monitored and we observed no differences between R6/1 and R6/1:TgRBFOX1 mice (Supplementary Fig. 5B).

We also decided to check whether correction of the Rbfox1 decrease of R6/1 mice, apart from attenuating motor phenotype, also has a positive impact on HD-associated neuropathology. For this, we first immunostained sagittal sections from 3.5-month-old WT, R6/1 and R6/1:TgRBFOX1 mice for the striatal marker DARPP32 to analyze atrophy (Fig. 3E) and for cleaved caspase 3 to detect apoptotic cells (Fig. 3F). This revealed that the decrease in striatal area observed in R6/1 mice respect to WT mice was significantly attenuated in R6/1:TgRBFOX1 mice. In good agreement, the significant increase in the number of cleaved caspase-3 positive cells detected in R6/1 mice is no longer observed in R6/1:TgRBFOX1 mice.

### RBFOX1 correction in HD mice attenuates mis-splicing of disease associated genes

**Fig. 4:**
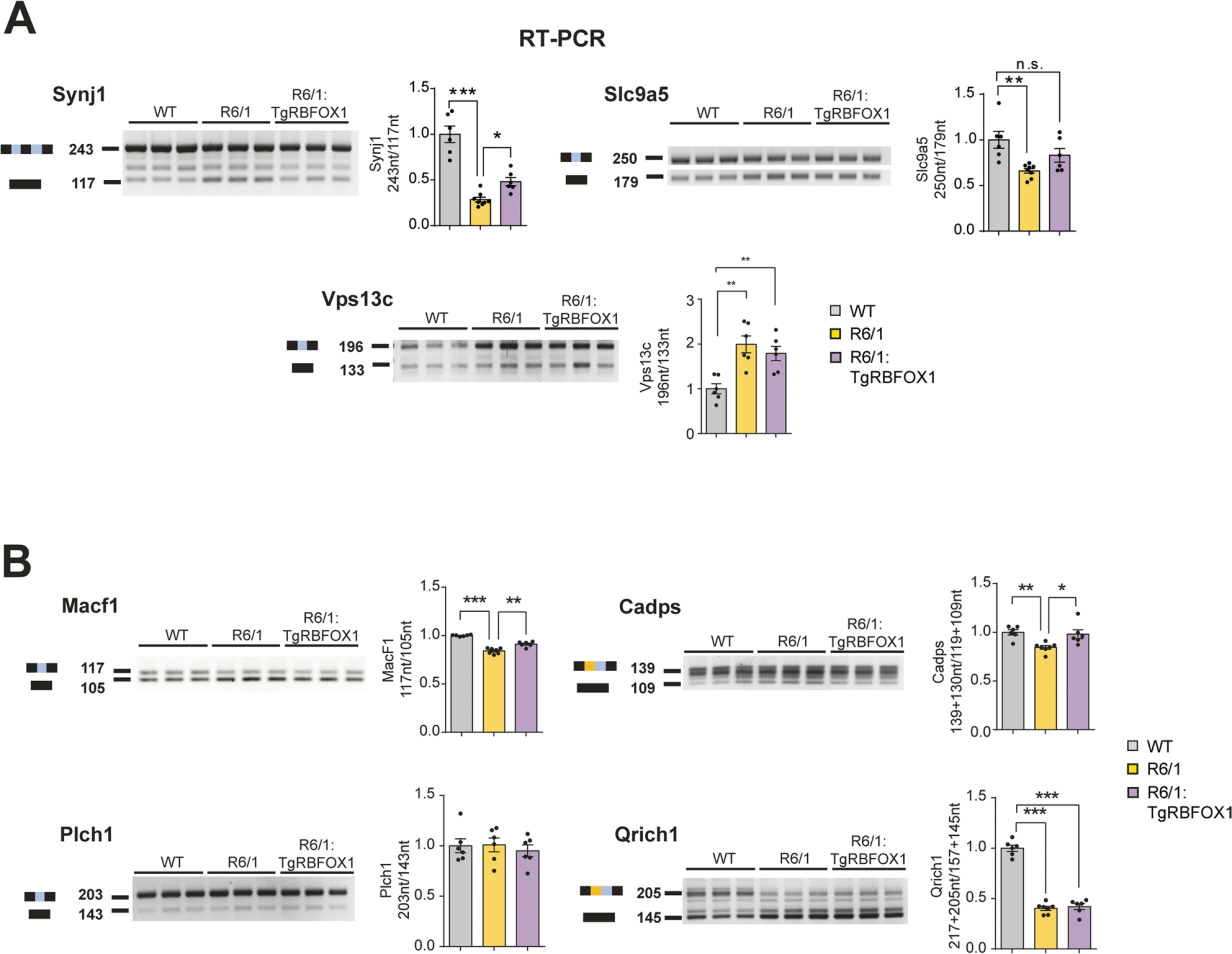
Overexpression of RBFOX1 nuclear isoform attenuates Huntington’s disease-associated mis-splicing in R6/1 mice. **A** Representative RT-PCR images and quantification of mis-spliced events of previously identified likely pathogenic effectors in cortex of 3.5-month-old WT (*n* = 6 [3 males and 3 females]), R6/1 (*n* = 7 [4 males and 3 females]) and R6/1:TgRBFOX1 (*n* = 6 [3 males and 3 females]) mice. **B** Representative RT-PCR images and quantification of prioritized RBFOX targets mis-spliced in cortex of 3.5-month-old WT (*n* = 6 [3 males and 3 females]), R6/1 (*n* = 7 [4 males and 3 females]) and R6/1:TgRBFOX1 (*n* = 6 [3 males and 3 females]) mice (ANOVA followed by Tukey’s or Games-Howell post hoc test; *P < 0.05; **P < 0.01; ***P < 0.001). Graphs show means ± SEM

In order to assess the amelioration of mis-splicing of RBFOX target exons, we identified transcripts that are both mis-spliced in HD and direct RBFOX targets. To identify the latter, we took advantage of data from a previous high-throughput sequencing-CLIP (HITS-CLIP) analysis that identified RBFOX-binding transcripts containing the (U)GCAUG motif (Weyn-Vanhentenryck et al. 2014). We then examined their representation among the 245 genes displaying altered cassette exon inclusion in the HD mis-splicing signature (Elorza et al. 2021). As expected, given the reported overrepresentation of the (U)GCAUG motif in the sequences flanking the HD mis-spliced exons (Elorza et al. 2021), we found an outstanding overlap, as one third of the HD mis-spliced genes correspond to direct RBFOX targets (RF: 8.0, *P* < 2.5 × 10–54) (Supplementary Fig. 6). We observed that, out of the six HD mis-spliced genes that were identified as likely pathogenic effectors in our previous study (Elorza et al. 2021), three —*Synj1*, *Slc9a5* and *Vps13c*— are direct RBFOX targets. Interestingly, by RT-PCR we noticed that the decreased exon inclusions observed in R6/1 mice respect to wild type mice for *Synj1* (contiguous exons) was attenuated in R6/1 mice with normalized Rbfox1 levels and (R6/1:TgRBFOX1 mice) and, for *Slc9a5* (cassette exon), the decreased inclusion respect to wild type mice was no longer significant in R6/1:TgRBFOX1 mice (Fig. 4A). These results suggest that RBFOX1 overexpression can be a good strategy to correct direct (motif containing) RBFOX target mis-splicing events with high pathogenic potential.

To test mis-splicing correction of additional likely pathogenic effectors, we generated a prioritized list of mis-spliced exons in R6/1 mice that are direct RBFOX targets and that have been associated with brain disorders (Supplementary Table-S3). At the top of the list we found exons in: *Cadps* (a neural protein involved in priming secretory vesicles in neurons and neuroendocrine cells, that has been associated with bipolar disorder (Sitbon et al. 2022)), *Qrich*1 (a transcription factor associated with Ververi-Brady developmental syndrome, OMIM 617982), *Plch1* (a phospholipase associated with Holoprosencephaly 14, OMIM 619895), and *Macf1* (a protein involved in microtubule stabilization that has been associated with brain lissencephaly-9, OMIM 618325). As shown in Fig. 4B, we could verify decreased exon inclusion in R6/1 mice by RT-PCR for *Cadps*, *Qrich1* and *Macf1*. Interestingly, regarding *Macf1* and *Cadps*, such decrease was found attenuated in R6/1:TgRBFOX1 (Fig. 4B).

Altogether, our results demonstrate that transgenic neuronal overexpression of RBFOX1 suffices to attenuate the mis-splicing of brain disease-associated genes, and the neuroanatomical and motor abnormalities observed in HD mice. This strongly suggests that the decrease of RBFOX1 levels that takes place in brains of HD patients and mice, has an impact on HD pathogenesis.

## Discussion

Here we generated transgenic mouse lines with neuronal overexpression of RBFOX1 to attenuate the decreased levels of this splicing factor in HD mice. The observed amelioration –although limited—, of molecular, histopathological, and behavioral phenotypes upon RBFOX1 overexpression demonstrates that diminished RBFOX activity contributes to HD pathogenesis, and suggests therapeutic potential of interventions able to increase RBFOX activity.

RBFOX1 was the best candidate member of its family of SFs to overexpress in HD mice because it is the first one to show decreased transcript and protein levels. However, along disease progression, the three members of the family show decreased protein levels, as we have previously reported (Elorza et al. 2021). Since we have only attenuated the deficit of Rbfox1, this may explain why only a minority of RBFOX-dependent events mis-spliced in R6/1 mice were found corrected in R6/1:TgRBFOX1 mice.

Understanding the mechanisms by which the levels of the three members of the RBFOX family decrease may help to envision ways to prevent such decrease. The early decrease of RBFOX1 protein levels can be explained by the also mentioned decreased transcript levels, but early decreases of RBFOX2 and RBFOX3 protein levels in striatum (Elorza et al. 2021), do not correlate with diminished transcript levels, suggesting that post-transcriptional mechanisms might also be at play. In this regard, CPEB4-dependent alterations in transcriptome polyadenylation and subsequent altered translation of affected transcripts has been reported in HD mice and, interestingly, Rbfox2 transcript is among those showing reduced polyadenylation in R6/1 mice (Picó et al. 2021), what is expected to contribute to decreased translation (Ivshina et al. 2014).

Another mechanism of posttranscriptional regulation of gene expression that is known to affect RBFOX genes and that might offer opportunities for therapeutic intervention, is the regulation of transcript stability and translation by microRNAs (miRNAs). At least three miRNAs have been shown to reduce RBFOX1 expression: miR-980 (Kucherenko and Shcherbata 2018) miR-129-5p (Rajman et al. 2017) and the Drosophila orthologue of human miR-9 (Katti et al. 2017). Neutralizing these miRNAs with antagomirs might have the desirable effect of increasing RBFOX protein levels. Antagomirs are a class of chemically engineered oligonucleotides designed to silence endogenous microRNAs as their sequence is complementary to their specific miRNA target (Stenvang et al. 2012). Interestingly, antagomirs have already been shown to be effective and safe in animal models of the trinucleotide repeat disorder myotonic dystrophy type 1 (DM1) (Cerro-Herreros et al. 2018). In that study, the authors sought to increase levels of a different splicing factor (MBNL) by antagonizing miR-23b and miR-218 and, as DM1 is a neuromuscular disorder, the target tissue was skeletal muscle. However, reaching the brain to increase RBFOX1 expression with potential miR-980 or miR-129-5p antagomirs can be more challenging as they should be engineered to cross the blood-brain barrier. It is worth noting though, that we have limited our study to analyzing RBFOX levels and functional implications in the brain of HD mice and patients but, since RBFOX-dependent AS is also important in muscle physiology (Singh et al. 2018) and this tissue is also affected in HD (Zielonka et al. 2014), we cannot fully discard a role of RBFOX also in HD peripheral tissues. However, exploring this goes beyond the scope of the present study.

Since we have observed microcephaly in the TgRBFOX1 mouse line with highest transgene expression (StrongTgRBFOX1 mice) and anticipation of the motor phenotype of R6/1 mice when these are combined with StrongTgRBFOX1 mice, this warns about a potential deleterious effect of excessive overexpression of RBFOX1. In line with this possibility, an increase in RBFOX1 levels –and an associated aberrant pattern of alternative RNA-processing— have been reported in neurons derived from iPSCs of Parkinson´s disease (PD) patients (Lin et al. 2016).

Interestingly, a study performed on an established isogenic HD cell model demonstrates widespread neuronal differentiation stage- and CAG length-dependent splicing changes (Mullari et al. 2023) and we noticed that the genes whose mis-splicing gets corrected by our RBFOX1 overexpression approach (Synj1, Macf1, Cadps and Slc9a5) are also found mis-spliced in the isogenic HD cell model, thus suggesting that many of the RBFOX1 dependent mis-splicing events might be taking place at early stages of neuronal maturation and prodromal disease. Curiously, two of these mis-splicing events (Macf1, Cadps) correspond to microexons, which are exons of 30 or fewer nucleotides with neural specific and neurodevelopment-associated alternative splicing patterns (Irimia et al. 2014, Gonatopoulos-Pournatzis and Blencowe 2020).

As already mentioned, the overall rescue of HD-associated molecular, histopathological, and behavioral phenotypes upon RBFOX1 overexpression is limited. Most likely, decreased RBFOX1 levels and activity is just one of the many molecular alterations that are triggered by the HD causing mutation and contribute to the pathogenesis. Other well stablished disease mechanisms include mutant huntingtin misfolding and aggregation, transcriptional dysregulation, mitochondrial dysfunction, oxidative stress, impaired proteostasis and protein degradation, disrupted axonal transport and synaptic dysfunction, and excitotoxicity and calcium dysregulation (Zuccato et al. 2010). Obviously, it was not expected that correction of RBFOX1 levels could overcome all the HD mutation–associated toxicities, but the amelioration observed in this preclinical study strongly suggests that RBFOX1 correction might be worth pursuing as adjuvant to other potential disease modifying strategies.

## Conclusion

Here we demonstrate that decreased RBFOX1 contributes to HD pathogenesis, as correction of RBFOX1 deficit in forebrain neurons by genetic manipulation of HD mice attenuates their mis-splicing, neuropathology and motor symptoms. This study therefore suggests that RBFOX increasing strategies may represent a new avenue for therapeutic intervention for HD.

## Supplementary Information

Supplementary Material 1.

Supplementary Material 2.

Supplementary Material 3.

Supplementary Material 4: Supplementary Figure 1. Independent antibody validation of early Rbfox1 protein decrease in HD mice. Rbfox1 protein levels were analyzed by Western blot in the cortex of 3.5-month-old WT (n = 7 [4 males and 3 females]) and R6/1 (*n* = 7 [4 males and 3 females]) mice using the Novus anti-Rbfox1 antibody. Quantification was normalized to β-actin. Data are presented as mean ± SEM. Student’s t-test, **P < 0.01. Supplementary Figure 2. Validation of transgene transcript expression and associated brain weight phenotypes in TgRBFOX1 mice. (A-B) RT-qPCR quantification of transgenic RBFOX1 transcript levels (A) and total RBFOX1 (B) transcript levels in the cortex of 1.5-month-old Control (n=5;4;4;4), MildTgRBFOX1 (n=5;4), and StrongTgRBFOX1 (n=5;5) (Student´s t test or Wilcoxon rank-sum test; **P <0.01; ***P <0.001). Data represent mean ± SEM. (C) Histogram showing brain weight of Control (n=5), MildTgRBFOX1 (n=7) and StrongTgRBFOX1 (*n*=7) mice (ANOVA, followed by Tukey’s post hoc test; **P < 0.01; ***P < 0.001). Data represent mean ± SEM. (D) Representative images of DARPP32-immunostained striatal area and its quantification in sagittal sections at two different lateral coordinates of the mouse brain for control (*n* = 6) and MildTgRBFOX1 (*n* = 4) mice at 3.5 months of age. (Student´s t test). Data represent mean ± SEM. Supplementary Figure 3. Locomotor activity and motor coordination evaluation in newly generated transgenic lines. (A–B) Quantification of ambulatory episodes, total distance traveled, resting time, and vertical activity in the open field test (A), and latency to fall in the accelerating rotarod (B) in control (n = 9; 4 males and 5 females) and StrongTgRBFOX1 (*n* = 7; 4 males and 3 females) mice at two different ages (2 months and 3.5 months). (C–E) Quantification of ambulatory episodes, total distance traveled, resting time, and vertical activity in the open field test (C), latency to fall in the accelerating rotarod at 2 months and 3.5 months (D) and grip strength at 5 months (E) in control (n = 10; 6 males and 4 females) and StrongTgRBFOX1 (n = 7; 3 males and 4 females) mice (Student´s t test or Wilcoxon rank-sum test). Data represent mean ± SEM. Supplementary Figure 4. Earlier onset of rotarod motor impairment in R6/1 mice harboring the StrongRbfox1 transgene. Quantification of latency to fall in the accelerating rotarod test at 3.5 and 5 months of age, normalized to 2-month performance, in Control (n = 9 [4 males and 5 females]), R6/1 (n = 10 [5 males and 5 females]), and R6/1:StrongTgRBFOX1 (*n* = 13 [5 males and 8 females]) mice. Upper panel shows all mice, middle panel shows only males and lower panel shows only females. Statistical analysis was performed using two-way ANOVA followed by Tukey’s post hoc test; *P < 0.05; **P < 0.01; ***P < 0.001. Data are presented as mean ± SEM. Supplementary Figure 5. Experimental breeding scheme and body weight assessment of behaviorally tested mice. (A) Diagram of the breeding strategy used to obtain littermates of the three experimental genotypes: Control, R6/1 and R6/1:TgRBFOX1 (boxed in red). The three required breeding types are identified with blue dashed-line rectangles and, in the progenies, the expected Mendelian ratios (theoretical percentages) for the resulting genotypes are shown. (B) Longitudinal analysis of body weight over time in the experimental groups. Body weight of the mice subjected to behavioral assessment was monitored biweekly from 5 to 23 weeks (Control [6 males and 4 females]; R6/1 [6 males and 4 females]; and R6/1:TgRBFOX1 [7 males and 5 females]). Statistical analysis was performed using two-way ANOVA followed by Tukey’s post hoc test. ‘*’ indicate differences between Control and R6/1 mice (*P < 0.05; ***P < 0.001), whereas ‘#’ symbols indicate differences between Control and R6/1:TgRBFOX1 mice (###*P* < 0.001). Data are presented as mean ± SEM. Supplementary Figure 6. Enrichment of RBFOX direct and functional targets among Huntington’s disease (HD) mis-splicing signature. Venn diagram showing the 83 genes in the intersection between the 245 genes with exons mis-spliced in HD (Elorza et al. 2021) and the 543 genes with RBFOX-direct target exons detected by HITS-CLIP (Weyn-Vanhentenryck, S.M., et al. 2014).

## Data Availability

Data generated in this study are available from the corresponding author upon reasonable request. The RNA-seq dataset will be available at the European Nucleotide Archive (ENA) database with accession number PRJEB110930.
