# Supplementary material for: Modest rescue of RBFOX1 splicing function attenuates Huntington’s disease features
Source: Mol Med. 2026 Apr 2;32:56. doi: 10.1186/s10020-026-01471-y (PMC13067610; doi:10.1186/s10020-026-01471-y)
Supplement: Supplementary file 4 — Supplementary Material 4: Supplementary Figure 1. Independent antibody validation of early Rbfox1 protein decrease in HD mice. Rbfox1 protein levels were analyzed by Western blot in the cortex of 3.5-month-old WT (n = 7 [4 males and 3 females]) and R6/1 (n = 7 [4 males and 3 females]) mice using the Novus anti-Rbfox1 antibody. Quantification was normalized to β-actin. Data are presented as mean ± SEM. Student’s t-test, **P < 0.01. Supplementary Figure 2. Validation of transgene transcript expression and associated brain weight phenotypes in TgRBFOX1 mice. (A-B) RT-qPCR quantification of transgenic RBFOX1 transcript levels (A) and total RBFOX1 (B) transcript levels in the cortex of 1.5-month-old Control (n=5;4;4;4), MildTgRBFOX1 (n=5;4), and StrongTgRBFOX1 (n=5;5) (Student´s t test or Wilcoxon rank-sum test; **P <0.01; ***P <0.001). Data represent mean ± SEM. (C) Histogram showing brain weight of Control (n=5), MildTgRBFOX1 (n=7) and StrongTgRBFOX1 (n=7) mice (ANOVA, followed by Tukey’s post hoc test; **P < 0.01; ***P < 0.001). Data represent mean ± SEM. (D) Representative images of DARPP32-immunostained striatal area and its quantification in sagittal sections at two different lateral coordinates of the mouse brain for control (n = 6) and MildTgRBFOX1 (n = 4) mice at 3.5 months of age. (Student´s t test). Data represent mean ± SEM. Supplementary Figure 3. Locomotor activity and motor coordination evaluation in newly generated transgenic lines. (A–B) Quantification of ambulatory episodes, total distance traveled, resting time, and vertical activity in the open field test (A), and latency to fall in the accelerating rotarod (B) in control (n = 9; 4 males and 5 females) and StrongTgRBFOX1 (n = 7; 4 males and 3 females) mice at two different ages (2 months and 3.5 months). (C–E) Quantification of ambulatory episodes, total distance traveled, resting time, and vertical activity in the open field test (C), latency to fall in the accelerating rotarod at 2 months and 3.5 mo [file 10020_2026_1471_MOESM4_ESM.pdf]

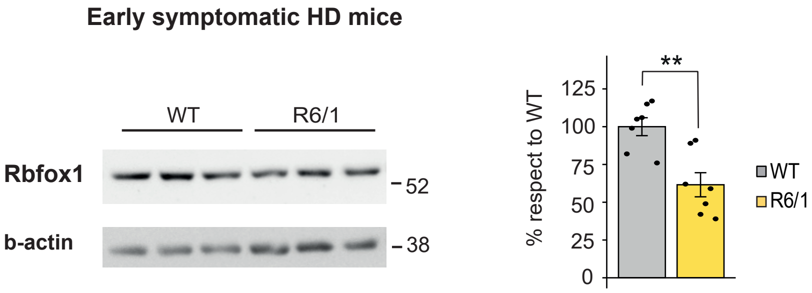

Supplementary Figure 1

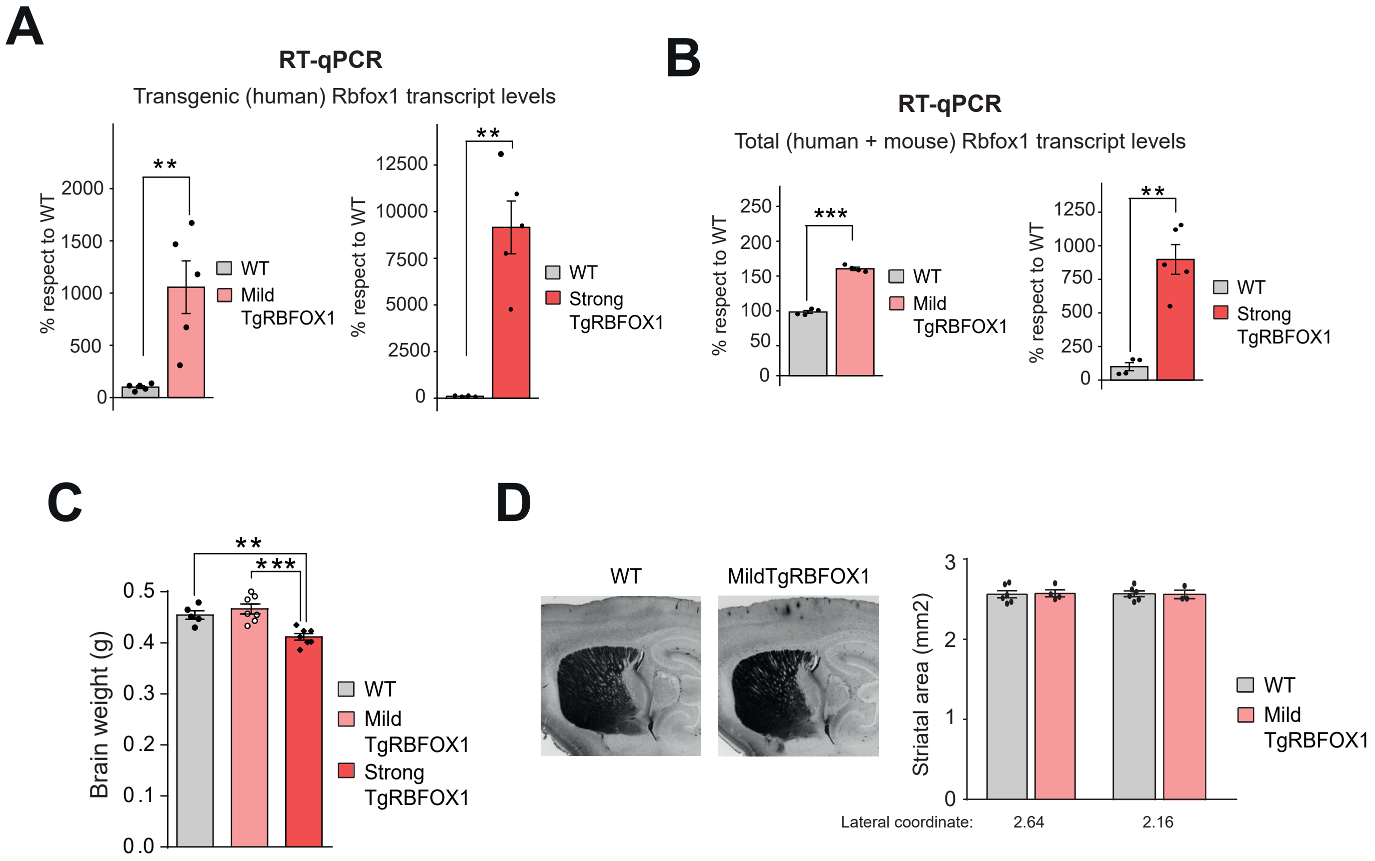

Supplementary Figure 2

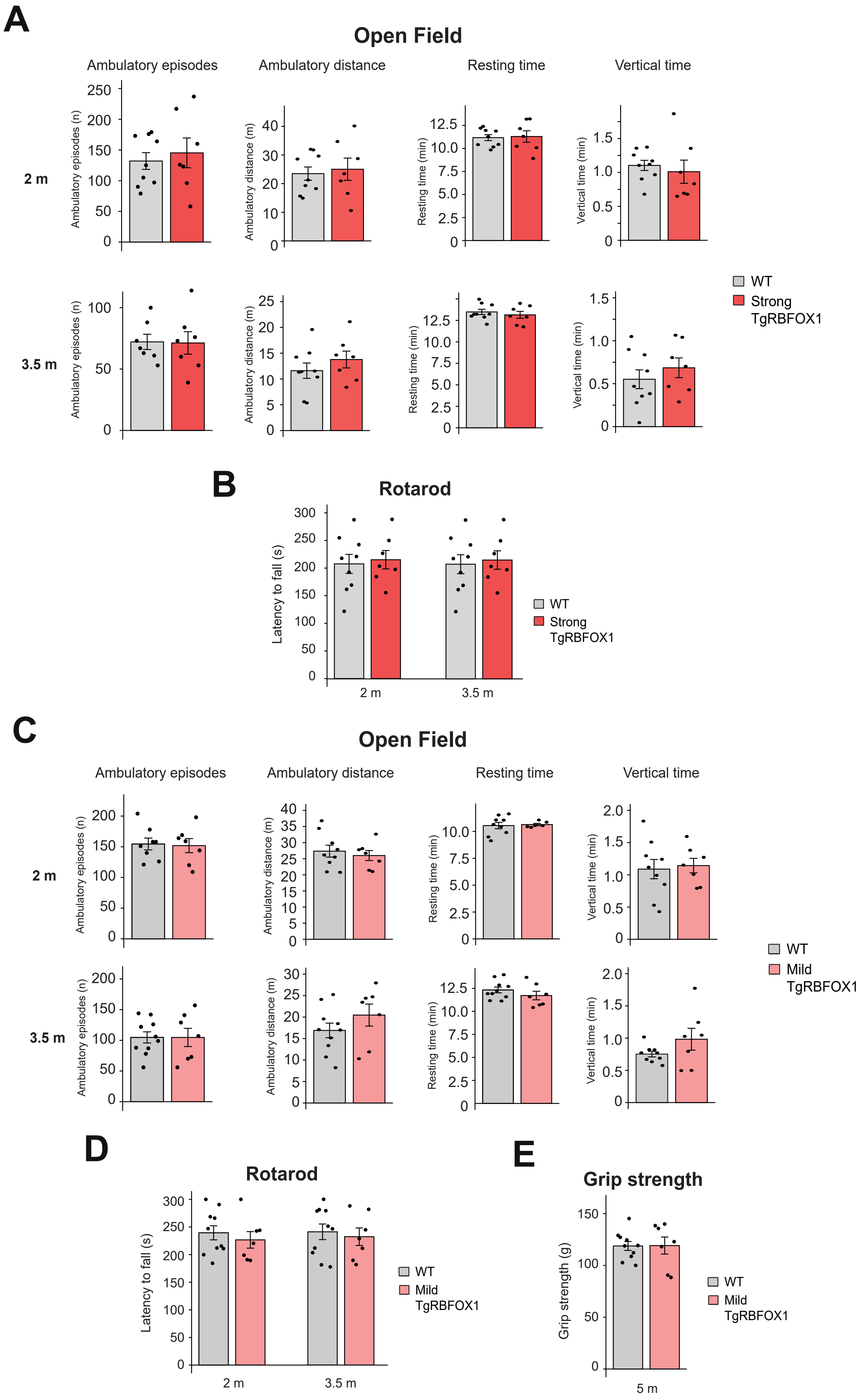

Supplementary Figure 3

R6/1:StrongTgRBFOX1

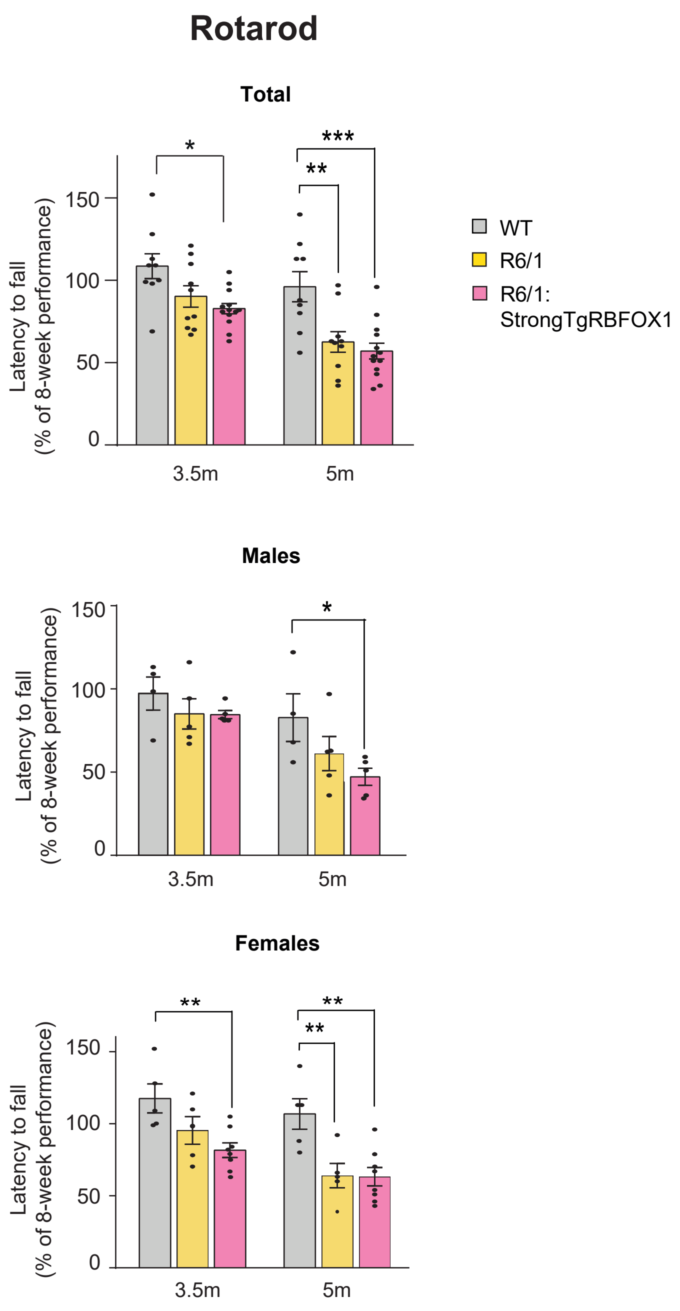

Supplementary Figure 4

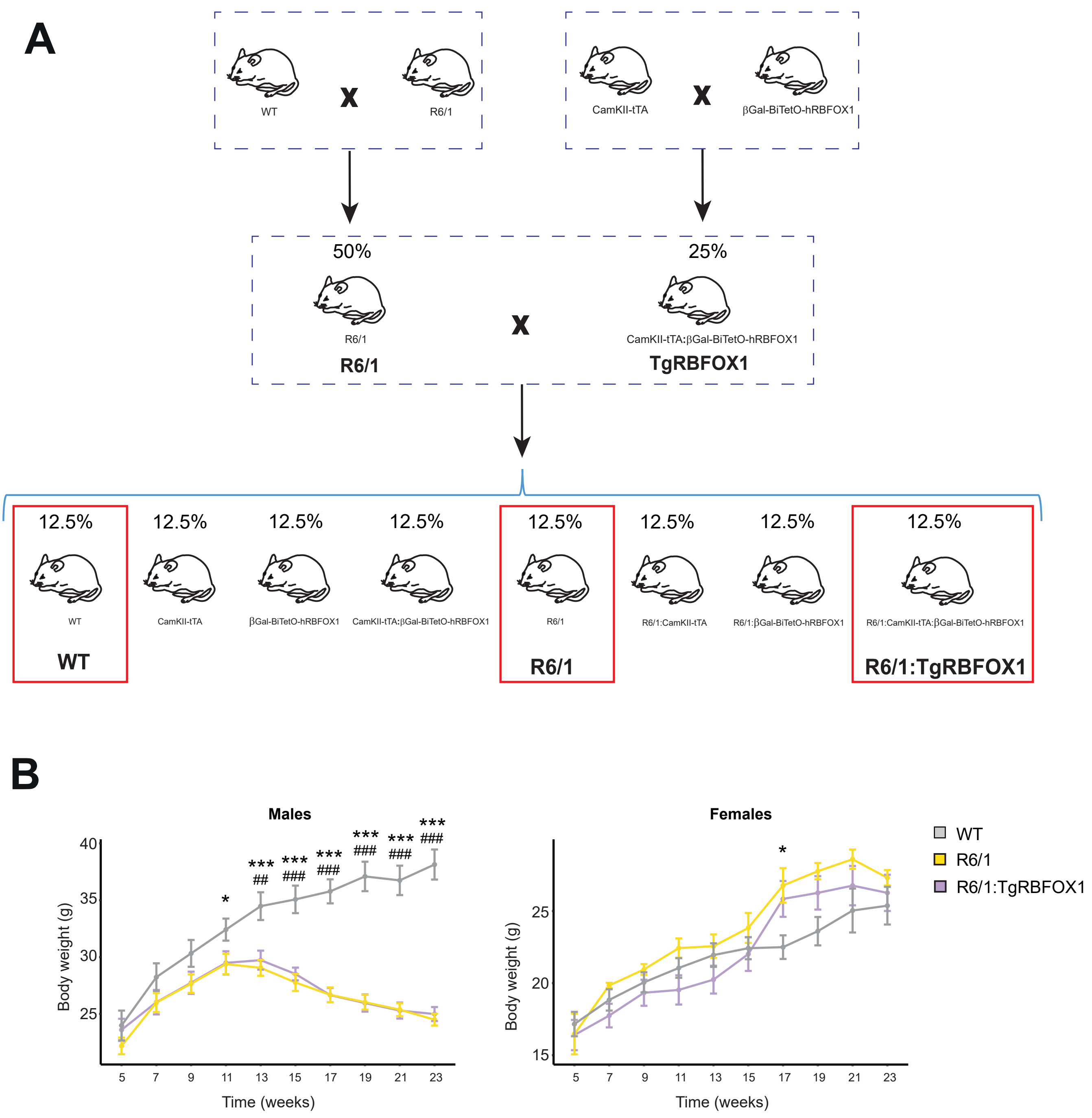

Supplementary Figure 5

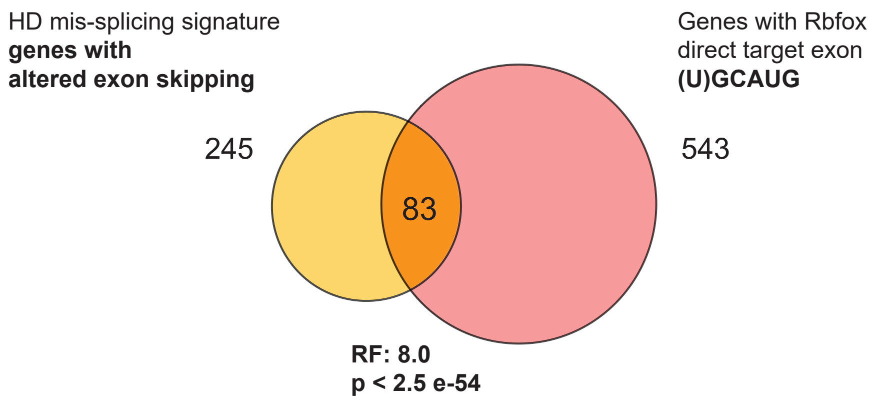

Supplementary Figure 6
